# Attenuated infection by a Pteropine orthoreovirus isolated from an Egyptian fruit bat in Zambia

**DOI:** 10.1371/journal.pntd.0009768

**Published:** 2021-09-07

**Authors:** Hayato Harima, Michihito Sasaki, Yasuko Orba, Kosuke Okuya, Yongjin Qiu, Christida E. Wastika, Katendi Changula, Masahiro Kajihara, Edgar Simulundu, Tomoyuki Yamaguchi, Yoshiki Eto, Akina Mori-Kajihara, Akihiko Sato, Satoshi Taniguchi, Ayato Takada, Masayuki Saijo, Bernard M. Hang’ombe, Hirofumi Sawa

**Affiliations:** 1 Hokudai Center for Zoonosis Control in Zambia, Research Center for Zoonosis Control, Hokkaido University, Sapporo, Japan; 2 Division of Molecular Pathobiology, Research Center for Zoonosis Control, Hokkaido University, Sapporo, Japan; 3 International Collaboration Unit, Research Center for Zoonosis Control, Hokkaido University, Sapporo, Japan; 4 Division of Global Epidemiology, Research Center for Zoonosis Control, Hokkaido University, Sapporo, Japan; 5 Department of Para-clinical Studies, School of Veterinary Medicine, the University of Zambia, Lusaka, Zambia; 6 Department of Disease Control, School of Veterinary Medicine, the University of Zambia, Lusaka, Zambia; 7 Macha Research Trust, Choma, Zambia; 8 Division of Bioresources, Research Center for Zoonosis Control, Hokkaido University, Sapporo, Japan; 9 Drug Discovery & Disease Research Laboratory, Shionogi & Co., Ltd., Osaka, Japan; 10 Department of Virology I, National Institute of Infectious Diseases, Shinjuku, Tokyo, Japan; 11 Africa Center of Excellence for Infectious Diseases of Humans and Animals, the University of Zambia, Lusaka, Zambia; 12 Global Virus Network, Baltimore, Maryland, United States of America; 13 One Health Research Center, Hokkaido University, Sapporo, Japan; Colorado State University, UNITED STATES

## Abstract

**Background:**

Pteropine orthoreovirus (PRV) is an emerging bat-borne zoonotic virus that causes severe respiratory illness in humans. Although PRVs have been identified in fruit bats and humans in Australia and Asia, little is known about the prevalence of PRV infection in Africa. Therefore, this study performed an PRV surveillance in fruit bats in Zambia.

**Methods:**

Egyptian fruit bats (*Rousettus aegyptiacus*, n = 47) and straw-colored fruit bats (*Eidolon helvum*, n = 33) captured in Zambia in 2017–2018 were screened for PRV infection using RT-PCR and serum neutralization tests. The complete genome sequence of an isolated PRV strain was determined by next generation sequencing and subjected to BLAST and phylogenetic analyses. Replication capacity and pathogenicity of the strain were investigated using Vero E6 cell cultures and BALB/c mice, respectively.

**Results:**

An PRV strain, tentatively named Nachunsulwe-57, was isolated from one Egyptian fruit bat. Serological assays demonstrated that 98% of sera (69/70) collected from Egyptian fruit bats (n = 37) and straw-colored fruit bats (n = 33) had neutralizing antibodies against PRV. Genetic analyses revealed that all 10 genome segments of Nachunsulwe-57 were closely related to a bat-derived Kasama strain found in Uganda. Nachunsulwe-57 showed less efficiency in viral growth and lower pathogenicity in mice than another PRV strain, Miyazaki-Bali/2007, isolated from a patient.

**Conclusions:**

A high proportion of Egyptian fruit bats and straw-colored fruit bats were found to be seropositive to PRV in Zambia. Importantly, a new PRV strain (Nachunsulwe-57) was isolated from an Egyptian fruit bat in Zambia, which had relatively weak pathogenicity in mice. Taken together, our findings provide new epidemiological insights about PRV infection in bats and indicate the first isolation of an PRV strain that may have low pathogenicity to humans.

## Author summary

Pteropine orthoreovirus (PRV) is a causative agent of acute respiratory illness in humans in tropical and sub-tropical regions in Southeast Asia. PRVs have been originally isolated from fruit bats, and it is assumed that PRVs spread to humans by both bat-to-human and human-to-human transmission. Recently, an PRV was also detected from a fruit bat in the Afrotropical region and might potentially cause an emerging infection of the bat-borne zoonotic virus in Africa. However, little is known about the prevalence of PRV infection in Africa. In this study, we demonstrated the high prevalence of PRV infection in bat populations in Zambia and isolated a new strain of PRV from Egyptian fruit bats. In addition, we found that the bat-derived PRV strain had lower pathogenicity in mice than a human-derived PRV strain isolated from a patient in Southeast Asia. Our findings provide new epidemiological information about PRV in fruit bats in the Afrotropical region and indicate the first isolation of an PRV strain that may cause attenuated infection in humans.

## Introduction

Pteropine orthoreovirus (PRV), classified as *Nelson bay orthoreovirus* species belonging to the genus *Orthoreovirus* in the family *Reoviridae* by the international committee on taxonomy of viruses (ICTV), is an emerging bat-borne zoonotic virus that causes severe respiratory illness in humans. The prototype of PRV, Nelson Bay strain, was first isolated from blood of a flying fox (*Pteropus poliocephalus*) in Nelson Bay, Australia in 1968 (S1 Table) [1]. Further, the first clinical case of PRV infection was reported in a respiratory disease patient with high fever in an Malaysian hospital in 2006 [2], and multiple PRV strains have since been detected from patients with respiratory illness in Malaysia and Indonesia [3–7]. Some reports indicated that some patients were exposed to bats prior to disease onset, suggesting transmission of PRV from bats to humans [2,3,5]. The PRV genome was detected in 17% (34/200) of oropharyngeal swab samples taken from patients with acute upper respiratory diseases in Malaysia in an epidemiological study [8]. In addition, antibodies to PRV were detected in serum samples of 13% (14/109) of the volunteers in Malaysia [2], 4.4% (12/272) of the patients who visited hospitals in Vietnam [9], and 0.8% (7/856) of the adult patients with fever in Singapore [10]. Thus, PRV infection in Southeast Asia may potentially become a serious public health issue.

PRVs are fusogenic, non-enveloped, icosahedral, segmented, double-stranded RNA (dsRNA) viruses. The genome of PRV consists of 10 segments divided into three size classes based on gel electrophoresis mobility shift characteristics, namely, three large segments (L1–L3), three medium segments (M1–M3), and four small segments (S1–S4) [11]. To date, 15 PRV strains have been isolated from six fruit bat species (*Pteropus poliocephalus*, *Pteropus hypomelanus*, *Pteropus vampyrus*, *Rousettus leschenaultia*, *Rousettus amplexicaudatus*, and *Eonycteris spelaea*) in Australia, Malaysia, Indonesia, China, and the Philippines (S1 Table) [1,12–17]. Serological study of PRV in the Philippines reported that 83% of the tested bats (70/84) had neutralizing antibodies to PRV, suggesting that PRV is a common infection in wild bat populations in Southeast Asia [16]. PRVs were also isolated from monkey feces collected in Thailand, raising the hypothesis that other mammals, apart from bats, may also play a role in PRV transmission to humans [18]. Recently, a partial PRV genome was detected in an Angolan soft-furred fruit bat (*Lissonycteris angolensis*) in Uganda *via* a metagenomic approach, suggesting the new geographic distribution of PRV outside of Asia and Australia [19]. However, the prevalence and pathogenicity of PRVs in Africa are still unknown.

Due to the public health interest and the importance of understanding the prevalence of PRV infection in Africa, genomic screening for PRV in fruit bats was performed in Zambia. Herein, we describe the characterization of a novel PRV strain, Nachunsulwe-57, isolated from one Egyptian fruit bat (*Rousettus aegyptiacus*) in Zambia. Additionally, sero-surveillance of PRV infection in the bat populations was conducted in Zambia using a neutralizing test for Nachunsulwe-57. Furthermore, pathogenicity of Nachunsulwe-57 in mice was analyzed in comparison with the Miyazaki-Bali/2007 strain isolated from a patient with respiratory illness in Southeast Asia.

## Methods

### Ethics statement

Collections of bat samples used in this study were performed with permission from the Department of National Parks and Wildlife, Ministry of Tourism and Arts of the Republic of Zambia (permit numbers MTA/NWP/8/27/1 and DNPW8/27/1; guidelines Act No. 14 of 2015). Animal experiments were performed with approval from the Animal Care and Use Committee of Hokkaido University, following the Fundamental Guidelines for Proper Conduct of Animal Experiment and Related Activities in Academic Research Institutions under the jurisdiction of the Ministry of Education, Culture, Sports, Science and Technology in Japan (permit number 20–0026). The mice were inoculated with virus-containing solutions under proper anesthesia, and all efforts were made to minimize any potential pain and distress. The mice inoculated with viruses were monitored daily during the study period, and a humane endpoint was introduced for all mice with >25% initial body weight loss as previously described [20]. All experiments were performed at the Biosafety Level-2 and 3 facilities at the Research Center for Zoonosis Control, Hokkaido University, following the institutional guidelines (permit number 20006–6).

### Cells

African green monkey kidney (Vero E6) cells and human embryonic kidney-derived 293T (HEK293T) cells were used in this study. These cells were maintained in Dulbecco’s Modified Eagle’s Medium (DMEM; Sigma-Aldrich, St. Louis, MO, USA) supplemented with 10% fetal bovine serum (FBS), 100 units/mL penicillin, and 100 μg/mL streptomycin at 37°C with 5% CO_2_.

### Bat samples

In 2017–2018, 47 cave-dwelling Egyptian fruit bats (*Rousettus aegyptiacus*) and 33 wild straw-colored fruit bats (*Eidolon helvum*) were captured with permission from the Department of National Parks and Wildlife, Ministry of Tourism and Arts, Zambia in Chongwe (15.6°S, 28.7°E) and Ndola (12.9°S, 28.6°E), respectively [21]. Bat species were identified based on morphological characteristics and nucleotide sequence analysis of the mitochondrial 16S rRNA and the cytochrome *b* genes, as previously described [22]. Collected tissue samples were stored at –80°C.

### Detection of PRV genome by RT-PCR

Total RNAs were extracted from colon homogenates using TRIzol-LS (Invitrogen, Waltham, MA, USA) or the QIAamp Viral RNA Mini Kit (Qiagen, Hilden, Germany) according to the manufacturers’ instructions. The PRV genome was examined by RT-PCR with pan-orthoreovirus primer sets targeting RNA-dependent RNA polymerase genes using total RNAs from colon tissues (S2 Table) [23,24]. After confirmation by gel electrophoresis, positive-PCR products were subjected to direct sequencing using the BigDye Terminator v3.1 Cycle Sequencing kit (Applied Biosystems, Foster City, CA, USA).

### Virus isolation

Colon homogenates that tested positive for the PRV genome in RT-PCR were inoculated onto Vero E6 cell cultures, followed by 1 h incubation at 37°C in 5% CO_2_ for virus adsorption. After removal of the inocula, the cells were washed twice with PBS and maintained in Eagle’s minimum essential medium (MEM; Nissui Pharmaceutical Co., Tokyo, Japan) containing 5 μg/mL trypsin, 0.3% bovine serum albumin, 2 mM L-glutamine, 4% antibiotic–antimycotic solution (Anti-Anti; Gibco, Waltham, MA, USA), and 1.0 mg/mL NaHCO_3_ at 37°C in 5% CO_2_. When the cells displayed a cytopathic effect (CPE), the culture medium was harvested, centrifuged at 1,750 × *g* for 5 min, and stored at -80°C.

### Growth kinetics of PRV

In this study, a new strain of PRV was isolated from Egyptian fruit bats in Zambia. For comparison studies, an PRV strain, Miyazaki-Bali/2007, isolated from a patient with acute respiratory infection was used [4]. PRV strains were propagated in Vero E6 cells in DMEM supplemented with 2% FBS at 37°C and 5% CO_2_. Virus titer was determined by the plaque assay using Vero E6 cells. Briefly, confluent monolayers of Vero E6 cells were infected with 10-fold serially diluted viruses. After adsorption for 1 h, the inocula were removed, and the cell monolayers were overlaid with DMEM containing 2% FBS and 0.7% agarose. Plaque-forming units (PFU) were determined three days post-infection (dpi).

Vero E6 cells were infected with Nachunsulwe-57 or Miyazaki-Bali/2007 at a multiplicity of infection (MOI) of 0.01. After adsorption for 1 h, the inocula were removed, and the cells were incubated with DMEM containing 2% FBS at 37°C and 5% CO_2_. The cultured media of infected Vero E6 cells were harvested at 8, 24, 48, and 72 h post-infection (hpi). Subsequently, these samples were centrifuged at 2,300 × *g* at 4°C for 5 min to remove the cell debris. Viral titers of the supernatants were determined by the plaque assay using Vero E6 cells.

### Electron microscopic examination of viral particles

The morphology of viral particles was assessed by a negative staining method using an H-7650 Hitachi transmission electron microscope (Hitachi High Technologies, Tokyo, Japan) as previously described [25]. Vero E6 cell monolayers were infected with the isolated virus, and the supernatants were ultracentrifuged at 174,000 × *g* and 4°C for 2 h. The concentrated viruses were resuspended in PBS, fixed with 0.25% glutaraldehyde, and negatively stained with 2% phosphotungstic acid solution (pH 5.8).

### Electrophoresis of viral dsRNA

The electrophoretic patterns of the viral dsRNA of the isolated virus were observed by sodium dodecyl sulfate–polyacrylamide gel electrophoresis (SDS-PAGE) as previously described [26]. Briefly, the supernatants of the infected cells exhibiting CPEs were ultracentrifuged for virus concentration. Total RNAs were extracted from the viruses and purified with LiCl precipitation. Subsequently, the purified viral dsRNA was separated by SDS-PAGE using 4–15% gradient gels and visualized *via* ethidium bromide staining.

### Full genome sequencing of the isolated virus

Complete genome sequences of the isolated virus from Egyptian fruit bats were determined by whole-genome sequencing and the rapid amplification of cDNA ends (RACE) method, as previously described [26]. Briefly, supernatants from the virus-inoculated cells were ultracentrifuged at 174,000 × *g* at 4°C for 2 h, and the concentrated virus was resuspended in PBS. Total RNA was then extracted from the concentrated virus using TRIzol-LS, and cDNA was synthesized using a PrimeScript Double Strand cDNA Synthesis Kit (Takara, Shiga, Japan) according to the manufacturer’s instructions. The double-stranded cDNA was fragmented, and index adapters were attached using a Nextera XT DNA Library Preparation Kit (Illumina, San Diego, CA, USA) according to the manufacturer’s instructions. The libraries were purified using AMPure XP (Beckman Coulter Inc., Brea, CA, USA) and quantified by a Qubit fluorometer with a Qubit dsDNA HS assay kit (Invitrogen) and an Agilent 2100 bioanalyzer with a High Sensitivity DNA kit (Agilent Technologies, Santa Clara, CA, USA), according to the manufacturer’s instructions. The prepared library was subsequently subjected to paired-end sequencing on a MiSeq instrument with a MiSeq Reagent Kit v3 (600 cycles) (Illumina). Sequence data were analyzed using the CLC Genomics Workbench software (CLC bio, Qiagen, Hilden, Germany). After trimming the low-quality reads, the remaining reads were *de novo* assembled and aligned back to the obtained contigs using the default setting. Obtained contigs were analyzed through a MegaBLAST search (https://blast.ncbi.nlm.nih.gov/Blast.cgi) with the default setting to identify the contigs of the viral genome. Consensus sequences with coverage of over 20 reads were obtained. Additionally, the 5′- and 3′-terminal regions of each segment were amplified by the RACE method with specific primers and a SMARTer RACE cDNA Amplification Kit (Takara), according to the manufacturer’s protocol (S3 Table). PCR products were subjected to direct sequencing using a BigDye Terminator v3.1 Cycle Sequencing kit (Life Technologies, Applied Biosystems, Foster City, CA, USA).

### Bioinformatic and phylogenetic analyses of PRV

Open-reading frame positions and encoded proteins were predicted through comparisons with other known PRV strains. The most similar sequence to each segment of the isolated PRV was identified through a MegaBLAST search with the default setting (https://blast.ncbi.nlm.nih.gov/Blast.cgi). Bioinformatic analyses were performed using various PRV sequences obtained from the GenBank database (S4 Table). Avian orthoreovirus was referred as an outgroup. Phylogenetic analyses based on the complete nucleotide sequence of each viral segment were performed using the MEGA7 software [27]. The MUSCLE protocol was used to align nucleotide sequences, and phylogenetic trees were constructed using the maximum likelihood method with the model of GTR+G+I for L1, L2, L3, M1 and S2 segments, the model of GTR+G for M2 and S1 segments, the model of HKY+G for M3 and S3 segments, and the model of K2+G for S4 segment as the best fit models, with 1,000 bootstrap replicates.

### Production of anti-PRV serum in guinea pigs

Five-week-old female Hartley specific pathogen-free (SPF) guinea pigs (Japan SLC Inc., Shizuoka, Japan) were used for the production of anti-PRV antiserum. The guinea pigs were intranasally inoculated with 2 × 10^6^ PFU of Nachunsulwe-57, followed by second and third intraperitoneal inoculations with 2.5 × 10^6^ PFU at one and three weeks after the first inoculation, respectively. Two weeks after the third inoculation, guinea pigs were sacrificed by exposure to excess isoflurane, and blood was collected. After centrifugation, serum containing anti-PRV antibodies was collected and stored at -80°C.

### Plaque reduction neutralization test

Serum samples collected from 70 of the 80 captured bats were used for the neutralization test. Bat and guinea pig sera were inactivated at 56°C for 30 min, and neutralizing antibody titers of the bat and guinea pig sera were examined by the 50% plaque reduction neutralization test (PRNT50). Each 50 μl of bat sera serially diluted 2-fold in PBS (1:20 to 1:5,120) was mixed with an equal volume of DMEM containing 100 PFU of Nachunsulwe-57 and incubated for 1 h at 37°C. As a negative control, viruses were mixed with an equal volume of 5% FBS diluted in PBS. The mixtures were inoculated onto confluent Vero E6 cells, followed by 1 h of incubation at 37°C in 5% CO_2_. After removal of the inocula, the cells were washed twice with PBS and overlaid with DMEM containing 2% FBS and 0.7% agarose. Thereafter, the cells were fixed with 10% neutralized formalin overnight and stained with crystal violet for visualization of plaques at 3 days after the overlay with agarose. The neutralization titer was defined as the reciprocal value of the highest serum dilution at which the number of plaques showed 50% reduction compared with those in the absence of serum.

### Experimental infection of mice

Viruses were prepared for experimental infection of mice as previously described [20]. Briefly, Nachunsulwe-57 and Miyazaki-Bali/2007 were propagated in HEK293T cells in DMEM supplemented with 5% FBS at 37°C and 5% CO_2_. The supernatant of infected cells was layered onto a 30% sucrose cushion in PBS and ultracentrifuged for virus concentration. The pellet containing the virus was resuspended in PBS, and the infectious virus titer was determined by a plaque assay.

Four-week-old female BALB/cCrSlc SPF (BALB/c) mice were purchased from Japan SLC Inc. (Shizuoka, Japan). The mice were anesthetized with an appropriate amount of isoflurane using the open-drop and nose-cone methods, and the condition of the mice was monitored. After the anesthesia treatment, the mice (n = 20) were intranasally inoculated with either Nachunsulwe-57 or Miyazaki-Bali/2007 strain with 1.0 × 10^5^ or 1.0 × 10^6^ PFU of each PRV in 20 μL of PBS (n = 5 in each group). As a negative control, PBS (20 μL) was intranasally inoculated into control mice (n = 5). The body weight of the mice was monitored daily for 14 days after inoculation.

To investigate the extent of viral replication in mouse lungs, six mice per group were intranasally inoculated with 1.0 × 10^5^ or 1.0 × 10^6^ PFU of each PRV as described above. The mice were sacrificed by exposure to excess isoflurane at 4 days after infection, and their lungs were collected in MEM containing 1% Anti-Anti. Collected lungs were homogenized using the BioMasher (Nippi, Tokyo, Japan) and disrupted by a freeze-thaw. The homogenates were centrifuged at 2,300 × *g* for 5 min to remove debris. Subsequently, the infectious virus titer in the supernatant fraction was determined by a plaque assay with Vero E6 cells. The detection limit of the virus titer was determined to be 10 PFU/0.1 g. The virus titers were plotted using the GraphPad Prism 8 software program (Graphpad Software Inc., San Diego, CA, USA).

### Histopathological and immunohistochemical analyses

BALB/c mice (n = 6) were intranasally inoculated with either the Nachunsulwe-57 (n = 3) or Miyazaki-Bali/2007 strain (n = 3) and sacrificed through inhalation of isoflurane at 4 dpi. Lung tissues were collected and applied for pathological examination, including Hematoxylin and Eosin (HE) staining and immunohistochemical (IHC) analysis, as previously described [28]. The lungs of infected mice, in which inflammation was observed, were used for pathological examination. As the negative control, mice were intranasally inoculated with PBS. The collected lungs were fixed in 10% buffered formaldehyde, and the sections were stained with HE for histopathology. The IHC analysis was performed using an inactivated polyclonal guinea pig anti-PRV serum, which was confirmed to neutralize the Nachunsulwe-57 infection *in vitro*, to detect the PRV antigen in the lungs. The sections were incubated in 0.3% H_2_O_2_ in methanol to quench endogenous peroxidase activity, and the viral antigen was detected using 100-fold diluted anti-PRV serum in 10% goat serum, the VECTASTAIN ABC HRP Kit (guinea pig IgG; Vector Laboratories, Burlingame, CA, USA), and the Histofine diamino benzidine substrate (Nichirei, Tokyo, Japan) according to the manufacturers’ instructions. The sections were counterstained with hematoxylin.

### Statistical analysis

Statistical analyses were performed using the Graphpad Prism 8 software program (Graphpad Software Inc.). Differences in neutralization titers of sera of Egyptian fruit bats were statistically analyzed by the Tukey test. Differences in viral titers of lungs of mice were statistically analyzed by the Mann-Whitney *U*-test. Survival curves of mice were statistically analyzed by the log-rank test. *P* values below 0.05 were considered significant.

## Results

### Isolation and characterization of novel PRV strain

**Fig 1:**
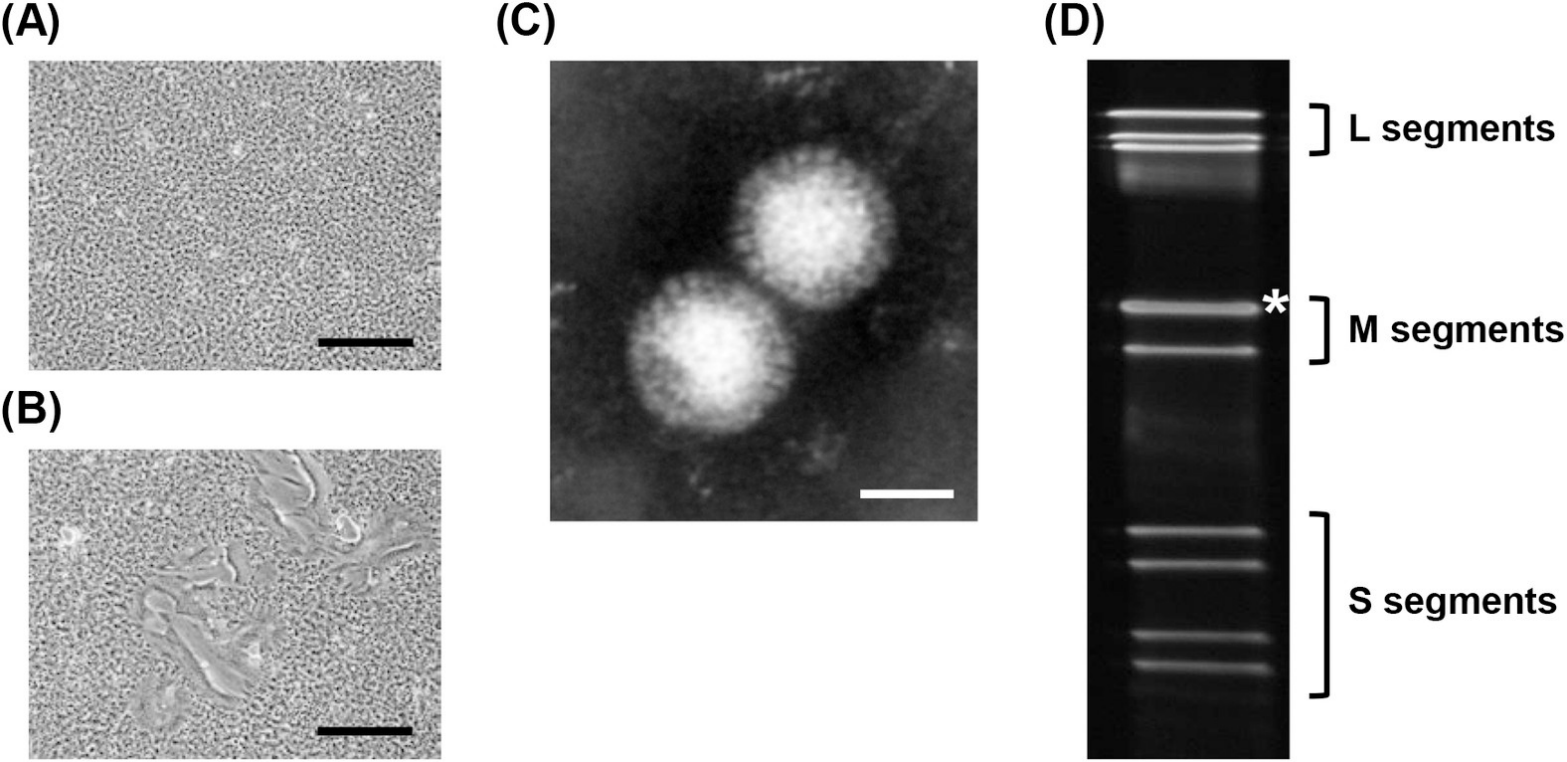
Isolation of PRV from bat colon specimen. (A and B) Phase-contrast images of mock-infected (A) and bat colon specimen–inoculated Vero E6 cells (B) at 5 dpi. CPE represented by syncytium formation was observed in Vero E6 cells inoculated with the bat colon specimen. Scale bar, 500 μm. (C) Electron micrograph of the virus isolated from the bat colon specimen. Scale bar, 50 nm. (D) Electropherotypes of viral dsRNA extracted from the isolated virus were observed by SDS-PAGE using 4–15% gradient gel. The locations of three L segments, three M segments, and four S segments are indicated. The M1 and M2 segments were considered to have co-migrated, forming a single band (*).

In total, 80 colon specimens collected from bats in Zambia underwent screening for the PRV genome by RT-PCR. Of these, the PRV RNA genome was detected in only one sample from an Egyptian fruit bat (*Rousettus aegyptiacus*; Bat ID, ZB18-57) captured in the Leopards Hill Cave in Chongwe (Table 1). The PRV genome-positive colon homogenate was inoculated onto Vero E6 cells for PRV isolation. After 3–4 days, clear CPE with syncytium formation was observed in the inoculated cells (Fig 1A and 1B). The cell supernatant was then examined by transmission electron microscopy (TEM), and the morphology of virions was found to be spherical, nonenveloped, and approximately 85 nm in diameter (Fig 1C). After examining the electrophoretic pattern of the viral dsRNA by SDS–PAGE, 10 viral segmented dsRNA of the isolated virus were observed (Fig 1D). These findings are consistent with characteristics of a fusogenic reovirus [11].

**Table 1:** Summary of bat samplings and screening results of RT-PCR and neutralization test.

Bat	Sampling place	Sampling date	No. of positive cases/No. of colons tested for PRV by RT-PCR	No. of positive cases/No. of sera tested by neutralization assay
Straw-colored fruit bat	Ndola	December 2017	0/33	32/33
Egyptian fruit bat	Chongwe	February 2018	0/22	16/16
	Chongwe	September 2018	1/25	21/21

Neutralization titers of 20 or higher were considered positive.

Bioinformatics analyses of nucleotide sequences obtained *via* whole genome sequencing and RACE revealed that the genome of the isolated virus consisted of 10 segments (3954, 3896, 3832, 2295, 2145, 1984, 1617, 1322, 1192, and 1185 bp in length), encoding 12 open-reading frames (λA, λB, λC, μA, μB, μNS, p10, p17, σA, σB, σC, and σNS; Table 2). The 5′- and 3′-untranslated regions ranged 12–30 and 35–92 bp in length, respectively. The isolated virus possessed consensus genome sequences of the 5′- and 3′-termini (5′-GCUU and UCAUC-3′, respectively) that were the same as other reported PRVs [4,12,16,29]. This newly isolated virus was tentatively designated as Nachunsulwe-57 (derived from the local name of the cave “Nachunsulwe”). The determined genomic sequences of Nachunsulwe-57 were deposited in the DNA Data Bank of Japan (DDBJ) under the accession numbers, LC619329-LC619338.

**Table 2:** Genome organization of Nachunsulwe-57.

Segment	Genome length (bp)	The position of ORF	Protein	Terminal sequence (5’-3’)	
L1	3,896	13–3,861	λC	5′-GCUU	UCAUC-3′
L2	3,832	18–3,797	λB	5′-GCUU	UCAUC-3′
L3	3,954	28–3,897	λA	5′-GCUU	UCAUC-3′
M1	2,295	14–2,206	μA	5′-GCUU	UCAUC-3′
M2	2,145	26–2,053	μB	5′-GCUU	UCAUC-3′
M3	1,984	29–1,924	μNS	5′-GCUU	UCAUC-3′
S1	1,617	27–314	p10	5′-GCUU	UCAUC-3′
		277–699	p17		
		611–1,582	σC		
S2	1,322	16–1,266	σA	5′-GCUU	UCAUC-3′
S3	1,192	29–1,132	σNS	5′-GCUU	UCAUC-3′
S4	1,185	31–1,116	σB	5′-GCUU	UCAUC-3′

### Genetic characterization of Nachunsulwe-57

To identify PRV strains genetically related to Nachunsulwe-57, BLAST analyses were performed using the sequences of each viral segment (Table 3). Nine of 10 segments (L1, L2, M1-M3, and S1-S4) of Nachunsulwe-57 were closely related to those of Kasama strain, which was detected in an Angolan soft-furred fruit bat (*Lissonycteris angolensis*) in Uganda [19]. The BLAST analysis based on the L3 segment of Nachunsulwe-57 revealed that Nachunsulwe-57 shared the highest nucleotide identity (93.6%) with Kampar strain identified in humans in Malaysia [3]. However, the partial nucleotide sequence of the L3 segment of Nachunsulwe-57 showed high sequence identity (99.3%) with the Kasama strain (706 nucleotides of its L3 segment (corresponding to positions 2544–3249) were lost). These results suggested the existence of a PRV strain highly related to Kasama strain in Zambia.

**Table 3:** BLAST analysis based on nucleotide sequences of each segment of Nachunsulwe-57.

Segment	Identity (%)	Strain	Host	Country	Accession No.
L1	99.3	Kasama	Bat	Uganda	MT505322
L2	99.2	Kasama	Bat	Uganda	MT505323
L3	93.6	Kampar	Human	Malaysia	JF342656
M1	99.2	Kasama	Bat	Uganda	MT505315
M2	99.0	Kasama	Bat	Uganda	MT505316
M3	97.7	Kasama	Bat	Uganda	MT505317
S1	96.7	Kasama	Bat	Uganda	MT505318
S2	97.3	Kasama	Bat	Uganda	MT505319
S3	99.2	Kasama	Bat	Uganda	MT505320
S4	97.4	Kasama	Bat	Uganda	MT505321

**Fig 2:**
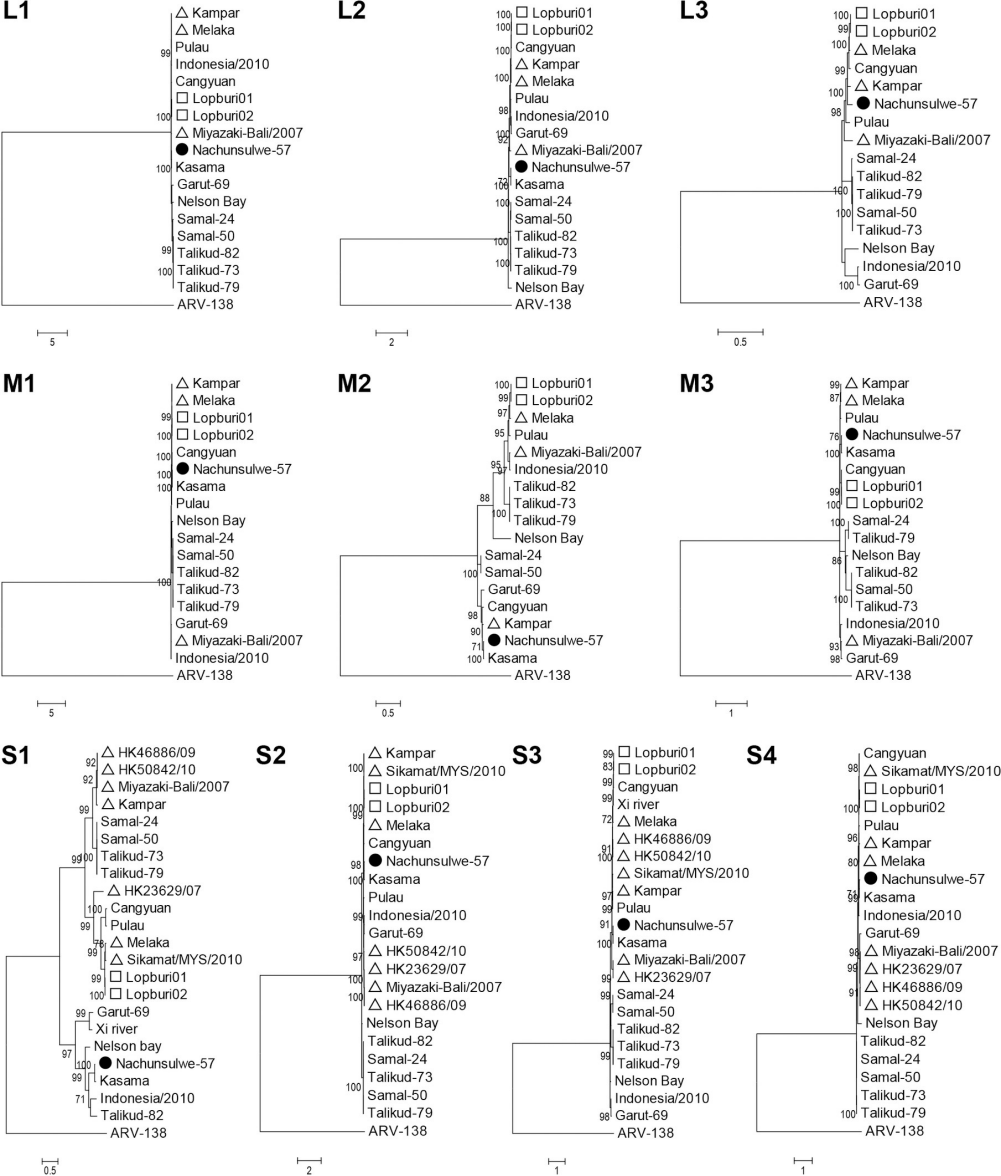
Phylogenetic analysis of 10 viral genome segments of PRV, including Nachunsulwe-57. Phylogenetic trees based on the nucleotide sequence of the complete L1-L3, M1-M3, and S1-S4 segments were constructed using the maximum likelihood method with 1,000 bootstrap replicates. The specie of *Avian orthoreovirus* was included as the outer group. Bootstrap values greater than 70% are shown on the interior branch nodes, and scale bars indicate the number of substitutions per site. Black circles represent the isolated PRV, Nachunsulwe-57. The triangles and squares represent PRV strains identified in humans and monkeys, respectively, while the other PRV strains were identified in bats. The Kasama strain was not included in the phylogenetic tree of L3 segment because the complete sequence of the Kasama strain L3 segment could not be obtained. Abbreviation: ARV-138, Avian orthoreovirus strain 138.

Phylogenetic trees of each viral segment were constructed using the nucleotide sequences of Nachunsulwe-57 and other PRVs (Fig 2). Based on the L1, L2, M1-M3, and S1-S4 phylogenies, Nachunsulwe-57 clustered only with Kasama strain. In the phylogenetic tree based on the L3 segment, Nachunsulwe-57 formed a branch near the Kampar strain without the Kasama strain because the complete genomic sequence of the L3 segment of the Kasama strain was not available. The two PRVs isolated in Africa did not form clusters with other PRVs identified in Asia and Australia.

### Seroprevalence of PRV infection in bats in Zambia and cross-reactivity of anti-PRV serum against PRV strains

The PRNT50 revealed that 98% (69/70) of bat sera had neutralizing antibodies against Nachunsulwe-57 with titers between 40 and 2,560, indicating remarkably high PRV seroprevalence in bats in Zambia (Tables 1 and S5). Sorting by species, 100% of Egyptian fruit bat sera (37/37) and 97% of straw-colored fruit bat sera (32/33) were positive for the neutralizing antibody. We further found that 38% of Egyptian fruit bat sera (14/37) had relatively high neutralization activities against Nachunsulwe-57, with serum titers of more than 1,000 and a mean value of 946. In contrast, sera of straw-colored fruit bats showed lower neutralization titers (mean value: 116). Considering the low positive rate of genomic detection for PRV (Table 1), these seroprevalences in both Egyptian fruit bats and straw-colored fruit bats are relatively high. These high seroprevalence against PRV might be due to the cross-reactivity of the examined serum to the PRV-related viruses.

**Fig 3:**
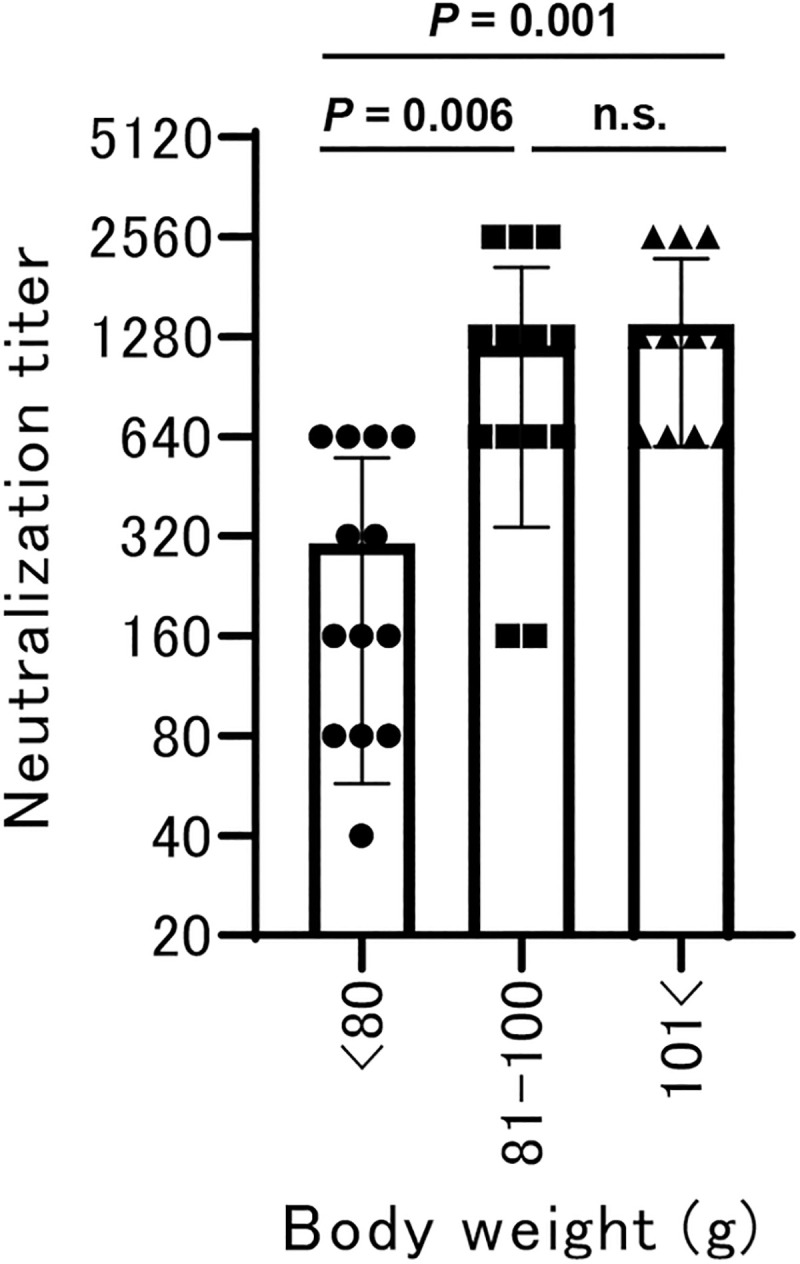
Neutralization titer of Egyptian fruit bats by body weight ranges. Egyptian fruit bats were classified into three group by body weight: juvenile group (<80 g, n = 13), small adult group (81–100 g, n = 13), and big adult group (101 g<, n = 11). Each plot represents the mean ± SD of 11 or 13 plotted neutralization titers. The indicated *P*-values were analyzed using the Tukey test. n.s., not significant.

To understand PRV infection related to the reproductive cycle of Egyptian fruit bats in Zambia, serological data were analyzed for body weight range. We divided the captured Egyptian fruit bats into three groups based on body weight as previously described [30]. These three groups of body weights <80 g, 81–100 g, and 101 g were designated as the juvenile, small adult, and big adult groups, respectively. We then compared the neutralization titers among these three groups (Fig 3). Interestingly, the juvenile group (<80 g) showed lower neutralization activities than both the small adult group (81–100 g) and the big adult group (101 g<). A serum collected from the bat actively infected with Nachunsulwe-57 (ZB18-57, 100 g) showed relatively low neutralizing activity, with a 160-neutralization titer. It is conceivable that juvenile bats are most likely to be actively infected with PRV, meanwhile most adult bats acquire sufficient immunity against to PRV infection. These results suggest that Egyptian fruit bats were commonly infected with PRV.

To evaluate cross-neutralization activity among PRV strains, PRNT50 was conducted using the anti-Nachunsulwe-57 serum obtained from guinea pigs inoculated with the virus. The serum from guinea pigs neutralized both Nachunsulwe-57 and Miyazaki-Bali/2007 infection, with neutralization titers of 40,960. This result indicated that the anti-Nachunsulwe-57 serum had cross-neutralization activity to the Miyazaki-Bali/2007 strain. Therefore, this polyclonal guinea pig anti-PRV serum was utilized for IHC analysis to detect PRV antigen in mouse organs.

### Virus replication of Nachunsulwe-57 in cell culture

**Fig 4:**
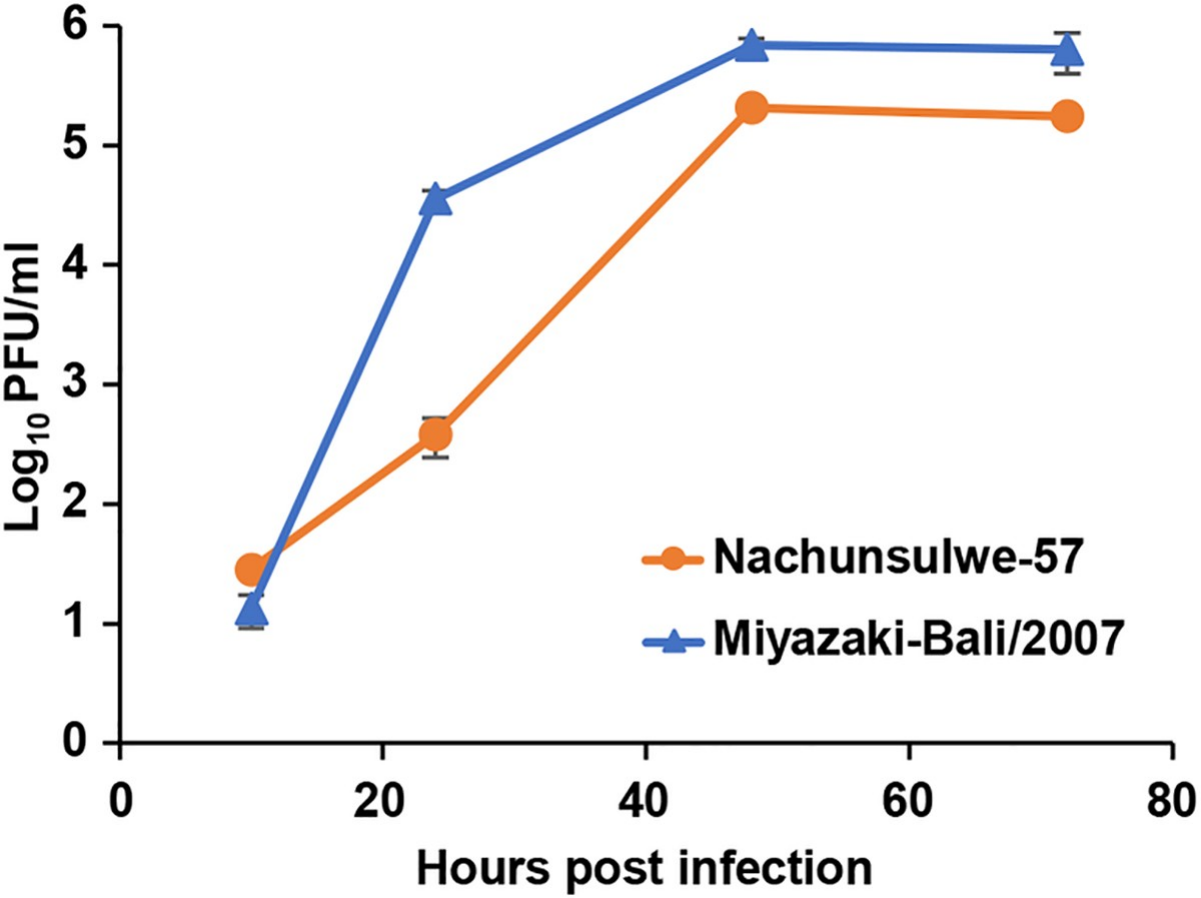
Growth kinetics of Nachunsulwe-57 and Miyazaki-Bali/2007 strain in cell culture. Vero E6 cells were infected with Nachunsulwe-57 or Miyazaki-Bali/2007 at 0.01 MOI. The supernatants were harvested at 10, 24, 48, and 72 hpi, and viral titers were determined by plaque assay using Vero E6 cells. Each value represents mean ± SEM of the results of three independent experiments.

Nachunsulwe-57 and Miyazaki-Bali/2007 replicated well in Vero E6 cells, and their viral titers nearly reached plateau levels at 48 hpi (Fig 4). However, viral growth of Nachunsulwe-57 was less efficient than that of Miyazaki-Bali/2007 in Vero E6 cells, and Nachunsulwe-57 had approximately 100-fold lower titers than Miyazaki-Bali/2007 at 24 hpi. Finally, Nachunsulwe-57 showed approximately 3-fold lower titers than Miyazaki-Bali/2007 at 48 and 72 hpi. These results indicated different biological phenotypes between these PRV strains.

### Pathogenicity of Nachunsulwe-57 in mice

**Fig 5:**
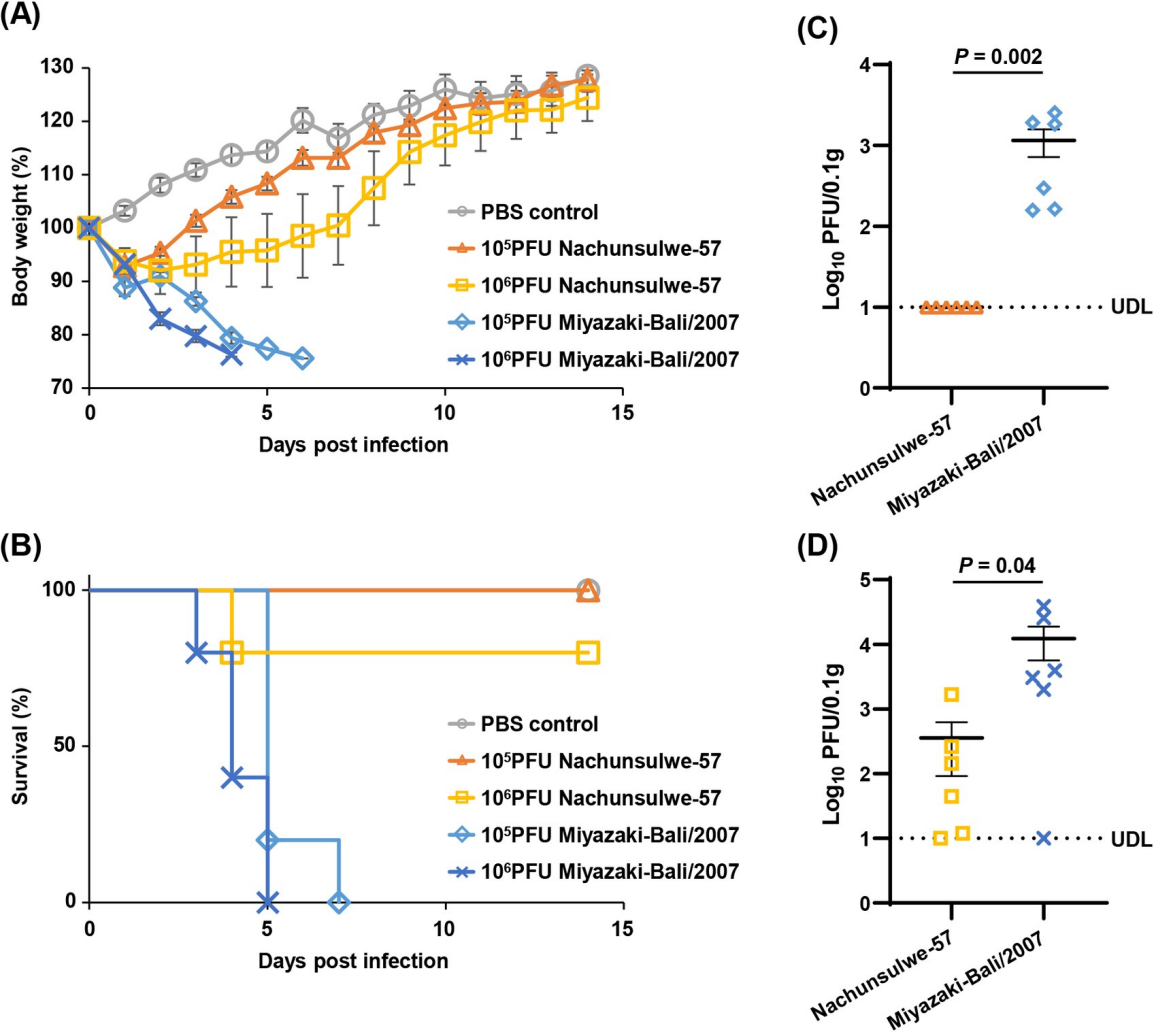
Pathogenicity of Nachunsulwe-57 and Miyazaki-Bali/2007 strains in mice. (A and B) Four-week-old female BALB/c mice were infected with Nachunsulwe-57 or Miyazaki-Bali/2007 at 10^5^ or 10^6^ PFU intranasally. Body weight (A) and survival rate (B) were monitored daily for 14 days. Each value represents mean ± SEM, n = 5. (C and D) At 4 dpi, viral titers in the lungs of BALB/c mice infected with Nachunsulwe-57 or Miyazaki-Bali/2007 at 10^5^ (C) and 10^6^ PFU (D) were examined. Each value represents mean ± SEM for each group, n = 6. The indicated *P*-values were analyzed by the Mann-Whitney *U* test. Dashed line indicates the limit of detection. UDL, under detection limit.

Pathogenicity of Nachunsulwe-57 was investigated using a mouse model of respiratory PRV infection, which was developed with Miyazaki-Bali/2007 in previous studies [20,31]. All of the mice intranasally inoculated with either 1.0 × 10^5^ or 1.0 × 10^6^ PFU of Miyazaki-Bali/2007 (five mice per group) showed weight loss at 1 or 2 dpi and died by 7 dpi (Fig 5A and 5B) as previously described [20]. In contrast, all mice inoculated with Nachunsulwe-57 at 1.0 × 10^5^ PFU (n = 5) survived with no body weight loss, except for a decrease at 1 dpi. In the group challenged with 1.0 × 10^6^ PFU of Nachunsulwe-57, three mice showed weight loss, although the other mice exhibited similar behaviors to those inoculated with 1.0 × 10^5^ PFU of Nachunsulwe-57. Finally, 20% mortality was observed in the group receiving 1.0 × 10^6^ PFU of Nachunsulwe-57. The survival curve was analyzed with the log-rank test, and the differences in the survival curves between mice inoculated with Nachunsulwe-57 or Miyazaki-Bali/2007 were statistically significant (1.0 × 10^5^ PFU, *P* = 0.0016; 1.0 × 10^6^ PFU, *P* = 0.0203).

Next, viral titers were examined in the lungs of mice inoculated with these viruses. In previous studies, the infectious virus was detected mainly in the nasal cavities, tracheas, and lungs, with lungs being a major site of PRV propagation [20,31]. At 4 dpi, the infectious virus of Miyazaki-Bali/2007 was detected in the lungs of mice (average level, 1.2 × 10^3^ PFU/0.1 g) inoculated with 1.0 × 10^5^ PFU of the virus (Fig 5C). In contrast, the infectious virus was not detected in the lungs of mice inoculated with Nachunsulwe-57, and the difference in viral titers in lungs of mice inoculated with Nachunsulwe-57 and Miyazaki-Bali/2007 was statistically significant (*P* = 0.002). Additionally, the viral titer of Nachunsulwe-57 in the lungs of mice inoculated with 1.0 × 10^6^ PFU was significantly lower than Miyazaki-Bali/2007 at 4 dpi (*P* = 0.04; Fig 5D). These results suggested that Nachunsulwe-57 was less pathogenic than Miyazaki-Bali/2007 in mice.

### Pathological examination in mice inoculated with PRV

**Fig 6:**
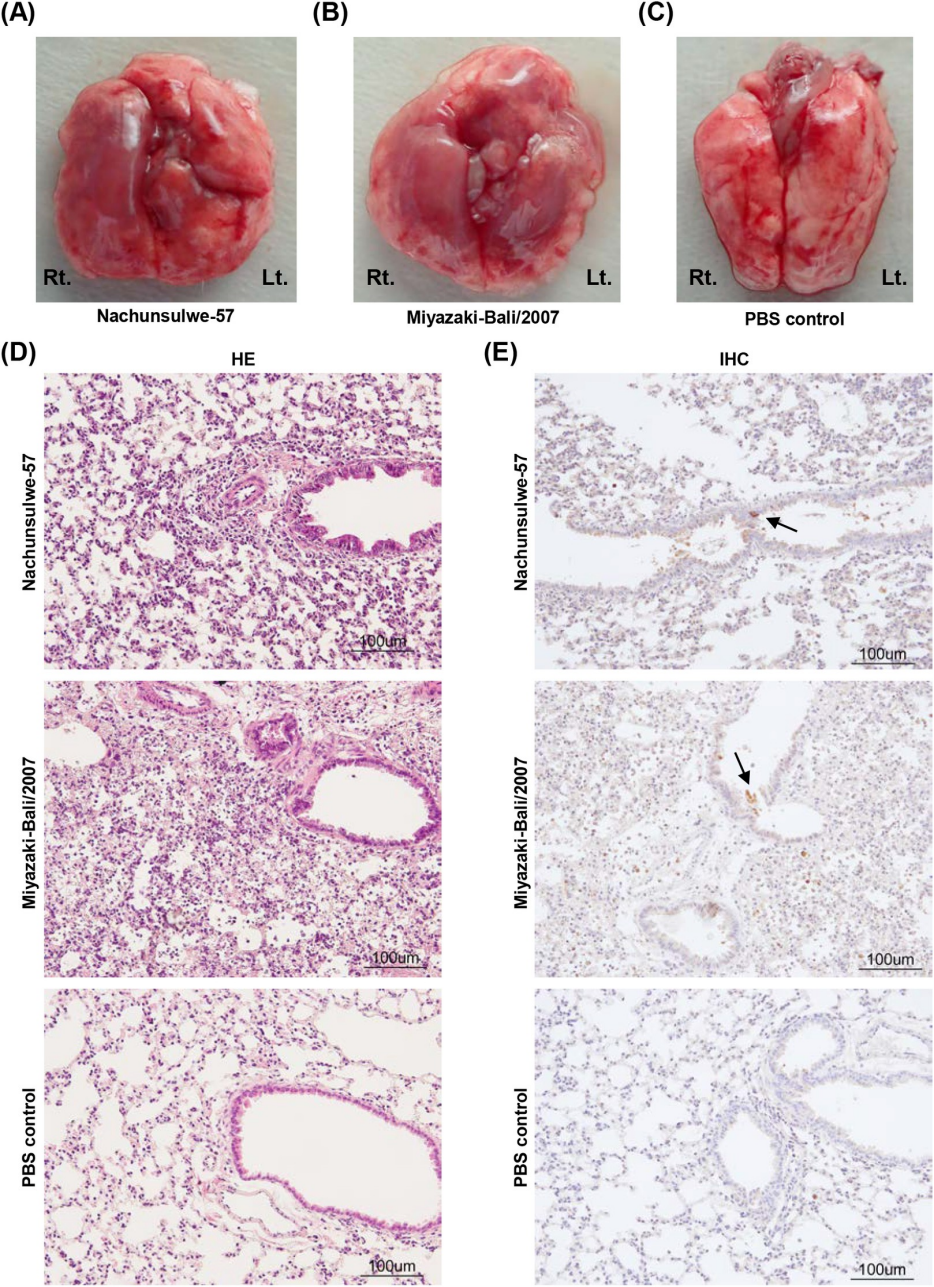
Pathological examination of BALB/c mice infected with Nachunsulwe-57 and Miyazaki-Bali/2007. The lungs were collected from BALB/c mice infected with Nachunsulwe-57 (10^6^ PFU; A) or Miyazaki-Bali/2007 (10^6^ PFU; B) at 4 dpi and control mice receiving PBS (C). Macroscopic images of the lungs were captured from the dorsal side. Lt, left lung; Rt, right lung. (D) HE staining images of the lungs of BALB/c mice at 4 dpi (magnification 200 ×, scale bars = 100 μm). (E) IHC analysis of the lungs at 4 dpi were performed using the anti-PRV polyclonal antibody. Black arrows indicate bronchiolar epithelial cells with immuno-positive signal for PRV (magnification 200 ×, scale bars = 100 μm).

Lung pathology of mice infected with Nachunsulwe-57 or Miyazaki-Bali/2007 at 1.0 × 10^6^ PFU at 4 dpi and control mice receiving PBS as a negative control was examined. Macroscopically, the lungs from mice infected with Nachunsulwe-57 and Miyazaki-Bali/2007 were swollen and discolored over the entire organ (Fig 6A–6C). Microscopically, peribronchial, perivascular, and interstitial accumulation of inflammatory cells with thickening of the intra-alveolar septum were detected in lungs of Nachunsulwe-57- and Miyazaki-Bali/2007-inoculated mice (Fig 6D). Furthermore, expression of the PRV antigen in lungs of the infected mice was evaluated by IHC analyses using the anti-PRV guinea pig serum. A small number of PRV antigen-positive cells were detected in the bronchial epithelium of mice infected with Nachunsulwe-57 or Miyazaki-Bali/2007 (Fig 6E). No positive signal was observed in PBS-inoculated control mice, demonstrating the specificity of the antibody used in the IHC analyses. No pathological change was macroscopically detected in other tissues, including liver, spleen, kidney, and intestine. Similar observations were reported about lung pathology of mice infected with Miyazaki-Bali/2007 in previous studies [20,31].

## Discussion

In this study, we have demonstrated the isolation of a novel PRV strain, tentatively named Nachunsulwe-57, from an Egyptian fruit bat in Zambia. We also characterized the Nachunsulwe-57 *in vitro* and *in vivo*. Although metagenomic analyses revealed the distribution of PRV in a fruit bat in Uganda [19], there is essentially no information about the prevalence of PRV infection in Africa. This is the first report of the PRV surveillance and isolation in fruit bats in Africa. We found that all segments of the Nachunsulwe-57 genome had highly shared identities (more than 96%) with the Kasama strain detected in Uganda, and that Nachunsulwe-57 clustered with the Kasama strain in the phylogenetic trees of PRV. These results suggest that the Nachunsulwe-57 strain seems to be a variant strain of the Kasama strain. Phylogenetic analyses also revealed that the African PRV strains were related to various PRV strains isolated from humans and bats in Asia and formed a lineage in each segment. Since other African strains have not been previously reported, African PRV strains may establish genetically distinct lineages in the African region. Complete genome sequences of all PRV segments are necessary to fully understand the evolutionary relationship among PRV strains. Further studies for determining the complete genome of various PRV strains in Africa and Asia are warranted to clarify the phylogeography of PRV. It is also worthy to note that two African PRV strains do not appear as a different group from the Asian PRV clades. As has been previously suggested, it is possible that PRV in Africa did not co-diverge with African fruit bats from their ancestral Asian lineages in the Miocene, but rather spread between African and Asian bats more recently [19]. Although our results support this suggestion, further epidemiological studies in Africa and Asia are required to understand the origin of PRV.

Although the PRV genome was detected only from one Egyptian fruit bats, 100% of sera collected from Egyptian fruit bats had neutralizing antibodies against PRV, indicating high prevalence of PRV infection in Egyptian fruit bats in Zambia. This high prevalence pattern was also observed in bat populations in the Philippines [16]. These results suggest that multiple species of fruit bats are commonly infected with PRV. Egyptian fruit bats are known to roost in caves, and the movements of Egyptian fruit bats are usually limited to less than 50 km [32, 33]. However, Nachunsulwe-57 is genetically related to Kasama strain identified in an Angolan soft-furred fruit bat in Uganda, at least 1,800 km apart from our sampling location. Therefore, PRV may spread by frequent contacts among multiple bat species via long-distance movement although the mechanism of PRV transmission between bat species remains unknown. PRV has also been isolated from crab-eating macaques in Thailand [18], and monkeys might act as an intermediate host for the cross-species transmission of PRV and its greater geographical distribution. It would be of interest to investigate PRV prevalence in wildlife, including in fruit bats and moneys, in African countries. It is also worth noting that juvenile Egyptian fruit bats showed lower neutralization activities than adult bats. In our previous study, a seasonal pulse of marburgvirus infection in Egyptian fruit bats in Zambia coincided with a single reproductive season of the bats, when the population of immunologically naive juvenile bats increased in the colonies [34]. As well as marburgvirus infection, there might be a seasonal pattern of PRV infection related to the reproductive cycle that is operational in Egyptian fruit bats in Zambia. This regular supply of naive bats through the reproductive cycles may partially explain how PRV is maintained in the Egyptian fruit bat population at a high level of seropositivity. However, the mechanism for PRV maintenance in its natural reservoir host is essentially unclear. Further epidemiological studies are required to understand the ecology of PRV in bat populations.

On the other hand, most of the straw-colored fruit bats tested in this study were positive for neutralizing antibodies for PRV, and their neutralization activities were lower than those of Egyptian fruit bats. Unlike Egyptian fruit bats, straw-colored fruit bats are an arboreal and migratory species with an extensive distribution across sub-Saharan Africa [32], and the potential interaction between these two bat species is unclear. Therefore, there is the possibility that this high seroprevalence against PRV was due to cross-neutralization activity among orthoreoviruses, and that the straw-colored fruit bats were not infected with Nachunsulwe-57, but with other PRV-related viruses. In our previous study, a novel orthoreovirus species was identified in Egyptian fruit bats in Zambia [24], but the cross-reactivity of neutralizing antibodies between these bat-derived orthoreoviruses remains unevaluated. Considering the unavailability of the genetic sequence of PRV in straw-colored fruit bats, the bats might harbor PRV-related viruses that elicits cross-reactive antibodies. Further epidemiological studies are required to clarify whether straw-colored fruit bats are infected with PRV.

The PRNT50 revealed that the anti-PRV serum obtained from Nachunsulwe-57-inoculated guinea pigs neutralized both Nachunsulwe-57 and Miyazaki-Bali/2007. In a previous study, the serum of a Miyazaki-Bali/2007-infected patient also had neutralization activities against other PRV strains, including Samal-24 and Talikud-82 [16]. These results suggest that the antibodies in the anti-PRV serum have cross-neutralization activity among multiple PRV strains. By intranasal PRV infection in mice, antibodies against 8 of 12 PRV proteins (λA, λC, μB, μNS, σA, σB, σC, and σNS) were generated [31]. Of these, antibodies to the σC, a cell attachment protein located at the surface of the virion, could mainly show neutralizing activities [11], while all the outer capsid proteins (λC, μB, and σB) might be targets of neutralizing antibodies. The σC protein shows large sequence divergence across PRV strains due to the immune pressure of neutralizing antibodies [11]. Although the σC of Nachunsulwe-57 was highly divergent from Miyazaki-Bali/2007 (amino acid identity, 41.1%), the serum of Nachunsulwe-57-inoculated guinea pigs showed high neutralizing activities against Nachunsulwe-57 and Miyazaki-Bali/2007, with neutralization titers of both 40,960. These results suggest that the σC of PRV is not the unique target of the neutralizing antibody, and a combination of antibodies against multiple PRV proteins may contribute to neutralizing activities against PRV.

Although the high prevalence of PRV infection in the bat populations in Zambia was clarified, no human case of PRV infection in Africa has yet to be reported. An influenza surveillance in Zambia in 2011–2017 revealed that influenza viruses were only detected in 8.7% of patients (894/10,212) with influenza-like illness or severe acute respiratory illness, implying that approximately 90% of patients with respiratory illness remained undiagnosed [35]. In another viral surveillance program in Zambia in 2018–2019, various respiratory pathogens, including influenza virus, respiratory syncytial virus, rhinovirus, and coronavirus, were detected in 63.2% of samples from 671 participants who presented influenza-like illness, suggesting that respiratory infections were associated with a wide range of viruses [36]. Further epidemiological investigation with additional diagnoses for respiratory pathogens, including PRV, is expected to identify the diverse etiologies of respiratory illnesses.

Finally, we evaluated the pathogenicity of Nachunsulwe-57 in a mouse model of PRV infection. Nachunsulwe-57 was found to be less pathogenic than Miyazaki-Bali/2007. In a previous study, the pathogenicity of both a human-derived strain (Miyazaki-Bali/2007) and bat-derived strain (Samal-24) was evaluated in mice, and Samal-24 caused viremia and respiratory disease similar to Miyazaki-Bali/2007, suggesting that the bat-derived PRV identified in Southeast Asia is potentially pathogenic to humans [20]. Conversely, the bat-derived PRV identified in Zambia may have low pathogenicity to humans and cause attenuated infection. Our findings may also be helpful to promote development of vaccines against PRV. According to the identity comparison of encoded proteins between Nachunsulwe-57 and Miyazaki-Bali/2007, all viral proteins except for those encoded in the S1 segment showed high amino acid sequence identities (more than 94.0%; S6 Table). The σC, p17, and p10 of Nachunsulwe-57 were highly divergent from those of Miyazaki-Bali/2007 (41.1%, 54.0%, and 75.7%, respectively). The σC and p10 are known to play an important role in viral pathogenesis in a mouse model [37,38] and may be related to attenuated infection by Nachunsulwe-57. Recently, a reverse-genetics system of PRV was established and enabled generation of mutant viruses affecting viral pathogenicity [37]. Further studies using this system with high- and low-pathogenic strains are required to clarify the mechanisms underlying the pathogenicity of PRVs.

## Supporting information

S1 Table Summary of PRV identified in humans, bats and monkeys. (XLSX)

S2 Table Primers used for PRV screening. (XLSX)

S3 Table Designed primers for RACE method. (XLSX)

S4 Table Reference sequences used in this study. (XLSX)

S5 Table Neutralization titers of bat sera for Nachunsulwe-57. (XLSX)

S6 Table Amino acid sequence identities of encoded proteins between Nachunsulwe-57 and Miyazaki-Bali/2007. (XLSX)
